# Web-Based Assessment of Visual and Visuospatial Symptoms in Parkinson's Disease

**DOI:** 10.1155/2012/564812

**Published:** 2012-03-20

**Authors:** Melissa M. Amick, Ivy N. Miller, Sandy Neargarder, Alice Cronin-Golomb

**Affiliations:** ^1^Translational Research Center for TBI and Stress Disorders, VA Boston Healthcare System, Boston, MA 02130, USA; ^2^Department of Psychiatry, Boston University School of Medicine, Boston, MA 02118, USA; ^3^Memorial Hospital of Rhode Island, Pawtucket, RI 02860, USA; ^4^Department of Psychology, Boston University, Boston, MA 02215, USA; ^5^Department of Psychology, Bridgewater State University, Bridgewater, MA 02325, USA

## Abstract

Visual and visuospatial dysfunction is prevalent in Parkinson's disease (PD). To promote assessment of these often overlooked symptoms, we adapted the PD Vision Questionnaire for Internet administration. The questionnaire evaluates visual and visuospatial symptoms, impairments in activities of daily living (ADLs), and motor symptoms. PD participants of mild to moderate motor severity (*n* = 24) and healthy control participants (HC, *n* = 23) completed the questionnaire in paper and web-based formats. Reliability was assessed by comparing responses across formats. Construct validity was evaluated by reference to performance on measures of vision, visuospatial cognition, ADLs, and motor symptoms. The web-based format showed excellent reliability with respect to the paper format for both groups (all *P*′*s* < 0.001; HC completing the visual and visuospatial section only). Demonstrating the construct validity of the web-based questionnaire, self-rated ADL and visual and visuospatial functioning were significantly associated with performance on objective measures of these abilities (all *P*′*s* < 0.01). The findings indicate that web-based administration may be a reliable and valid method of assessing visual and visuospatial and ADL functioning in PD.

## 1. Introduction

Visual and visuospatial deficits are common in Parkinson's disease (PD) and negatively affect everyday functioning. The PD Vision Questionnaire was developed to document the prevalence of these impairments [1]. It revealed that the large majority of respondents in the mild to moderate stages of the disease endorsed at least one such symptom [2]. Numerous studies have shown that individuals with PD demonstrate visual and high-order spatial impairments on laboratory-based assessments that cannot be accounted for by motor or executive dysfunction. Visual impairments and in particular, reduced contrast sensitivity, are well established [3–7]. In regard to visuospatial abilities, PD patients are impaired on global/local processing [8], a skill independent of executive demands, as well as mental rotation, way finding, visual construction, visuospatial reasoning, and angle size estimation [9–12]. Visual deficits have been linked to freezing of gait, an extremely debilitating motor symptom [2]. Further, PD-related visual and spatial abilities are predictors of the ability to drive, a visually mediated ADL [13–15] that is important to independent living. Considering their prevalence and negative functional impact, there is a critical need for further information on these underappreciated nonmotor symptoms.

A challenge to the assessment of a variety of aspects of PD is the burden of research participation when it must occur outside of an individual's home. Frequent office visits and distant travel have been identified as barriers to patient participation in PD research studies [16], raising the question of possible sample bias with respect to those individuals who have the time, motivation, and travel-related mobility to participate. Emerging data support the feasibility of Internet-based experimental tools for data collection from individuals with PD. Internet-administered experimental tasks have been developed and found comparable to office- or laboratory-based assessments for a variety of disorders [17–19]. Although tremor, rigidity, and bradykinesia might be expected to impact computer use, in fact many people with PD use the Internet for socialization, information gathering, and leisure activities despite their motor symptoms [20]. One of the benefits of online assessment is the potential to enroll larger numbers of research participants and include a wider range of participants (in terms of age and motor severity) than are usually assessed.

In order to assess visual and visuospatial impairments, we created a web-based version of our vision questionnaire. To evaluate the feasibility, reliability, and construct validity of the online version, we included this measure in a broad assessment of visual perception, cognition, and daily functioning in PD.

## 2. Materials and Methods

### 2.1. Participants

Twenty-four individuals with PD (12 women), recruited from the outpatient clinic of the Parkinson's Disease Center in the Department of Neurology, Boston Medical Center and from Boston-area PD support groups, took part in the study as well as 23 healthy control adults (HC) (16 women) who were community volunteers. Methods were approved by the Institutional Review Board (IRB) of Boston University and the Committee on Research with Human Subjects at Memorial Hospital of Rhode Island. Written informed consent was obtained from participants prior to their inclusion.

The PD and HC groups did not differ with respect to age, education, or male : female ratio (Table 1). There were no group differences in binocular near or far acuity (near acuity: *χ* ^2^ [6] = 4.5, *P*′*s* = 0.50; far acuity: *χ* ^2^ [6] = 4.1, *P*′*s* = 0.60). Near acuity was 20/25 (median) for both groups and far acuity was 20/20 (median) for both groups. No participant was demented as indexed by scores on the modified Mini-Mental State Examination (mMMSE) [21]. A cutoff score of 27 was used for HC participants. A score of 25 was used for PD participants because this form of the MMSE is particularly sensitive to specific cognitive deficits found in PD without dementia, as it includes tasks assessing executive functioning (scores converted from a 57-point scale). Participants were interviewed about their medical history, including ophthalmologic health, to rule out other confounding and exclusionary diagnoses such as stroke, head injury, serious medical illness, and ocular/optical abnormalities. The majority of participants (18 PD, 21 HC) underwent a detailed neuro-ophthalmological examination to confirm the absence of ocular disease. Participants who did and did not complete the neuro-ophthalmological examination did not differ in regard to participant characteristics or results.

Diagnosis of idiopathic PD, side of disease onset, and disease duration were confirmed by medical history and assessment with the Unified Parkinson's Disease Rating Scale (UPDRS). Two participants were not available for UPDRS administration. A Hoehn and Yahr (H&Y) score for stage of motor disability was derived from the UPDRS motor section [22]. Participants were in the mild to moderate stages of disease severity (Stage *1* = 2; Stage *2* = 3; Stage *2.5* = 11; Stage *3* = 6). All PD participants were taking medication for their motor symptoms and were tested when motor response was at its optimum (“on” period). Of the 24 participants, 23 followed a medication regimen that included levodopa/carbidopa therapy (*n* = 5), levodopa/carbidopa in combination with a dopamine agonist (*n* = 16), or dopamine agonist only (*n* = 2). One participant was not taking levodopa/carbidopa or a dopamine agonist. Group characteristics are summarized in Table 1.

### 2.2. Assessment

#### 2.2.1. PD Vision Questionnaire

Participants completed the 73-item Vision Questionnaire [2] twice: once in the standard paper format and once online. The paper format was administered first to all but five individuals for scheduling reasons. These five did not differ from the others in regard to participant characteristics or results. The average duration between repeat assessment was 1.5 months (SD: 1.2 months). Exact dates were not available for the paper questionnaires of three participants but the surveys were returned within about one month of the online assessment.

The online version of the questionnaire included several design features to improve Internet accessibility for participants with PD. Visual feature enhancement included large font (*∼*0.5 cm in height) and an option for increased contrast (negative polarity, white lettering on a black background), as some have found this to enhance computer screen reading [19]. Drop-down options were oversized to reduce issues with manipulating the mouse due to tremor or rigidity (>2 cm × 0.5 cm). To minimize the cognitive demands of the questionnaire, the format of each page was kept consistent in form, size, and location of information, and only one question was presented per page.

The PD Vision Questionnaire comprises three sections: *Visual and Visuospatial *symptoms, performance of visually mediated *Activities of Daily Living*, and *Motor* symptoms. Qualitative responses were not included in these analyses. Follow-up questions were not administered if the participant denied experiencing that particular symptom.

In order to evaluate the reliability and validity of the three content areas, summary scores for each section of the survey were created. Within each content area, severity ratings (Likert scale) of all questions administered to every participant were summed. The Visual and Visuospatial summary score comprised 14 items rated on a scale from one to nine, with “1” indicating the absence of the problem. The Motor summary score comprised 21 items rated on a 9-point scale. The ADL summary score comprised two scale items and 11 yes/no items reflecting the presence or absence of a problem (score of 1 for “yes” and zero for “no”). Seven of the ADL questions also had a “not applicable” response option, which was scored as zero. While this may underestimate the severity of ADL impairment, this approach was preferred to methods that might overestimate the degree of ADL impairment (e.g., number of responses endorsed positively divided by the number of questions answered). For six PD participants, one or more of the summary scores from the paper format of the questionnaire could not be calculated because of skipped responses to multiple items within a content area (Visual and Visuospatial *n* = 1, ADL *n* = 1, Motor *n* = 4). For PD participants with only a few missing responses, values were replaced with the mean for the PD group (3 or fewer respondents per item). For the paper questionnaire, each of the summary scores was calculated for the PD group whereas in the control group only the Visual and Visuospatial summary score was calculated, as most of the motor and activities of daily living items were not applicable to this group. In Section 3, reliability of the Visual and Visuospatial summary scores are reported for the control as well as the PD group.

#### 2.2.2. Construct Validation

Visual abilities included contrast sensitivity and coherent motion perception [7]. Contrast sensitivity was assessed with the chart-based Functional Acuity Contrast Sensitivity Chart (FACT) (Vision Sciences Research Corp., San Ramos, CA) and a computer-based backwards masking task [7]. Visuospatial abilities assessed included determination of the midpoint of a horizontal line with the Landmark Test of Line Bisection [23], map reading with the Standardized Road-Map Test of Direction Sense [24], and angle size estimation with the Benton Judgment of Line Orientation Test (JLO) [25]. Subjective quality of life and activities of daily living were assessed with the Parkinson's Disease Quality of Life Questionnaire-39 (PDQ-39) [26]. Motor symptoms were evaluated with the UPDRS [27].

### 2.3. Statistical Analysis

Group differences on continuous measures (e.g., age, education) were evaluated using *t*-tests, and on categorical variables using the chi-squared test. The reliability of the web-based survey (versus paper) and construct validity of the online survey were evaluated with Pearson correlations. Because many visual and visuospatial measures were used, composite scores were created to evaluate the construct validity of the visual and visuospatial summary score. *Z*-scores were derived for performance on vision tasks (Vision *z*-score: FACT contrast sensitivity [average log sensitivity across the five spatial frequencies], backwards masking [grey level] contrast sensitivity, and coherent motion perception [% coherent dot motion]), visuospatial tasks (Visuospatial *z*-score: Line Bisection [% of line], Road Map [total errors], JLO [total errors]), and the combined visual and visuospatial tasks (Visual and Visuospatial *z*-score), which were compared with the Visual and Visuospatial summary score from the Vision Questionnaire. Means (SDs) on the individual tests were as follows for PD and HC: FACT: PD: 1.5 (0.3), HC: 1.6 (0.2); backwards masking contrast sensitivity: PD: 124.8 (46.6), HC: 92.1 (8.1); motion perception: PD: 9.1 (4.8), HC: 8.4 (4.3); Line Bisection: PD: 14.4 (8.8), HC: 13.07 (5.7); Road Map: PD: 2.5 (4.0), HC: 3.1 (3.9); JLO: PD: 5.8 (4.3), HC: 4.6 (4.2). Because clinical and cognitive symptoms may differ in men and women with PD [28] and in PD patients with left versus right side of motor onset [10], we examined the results along these dimensions. Summary scores for the three construct areas did not differ with respect to gender (all *P*′*s* > 0.70) or body side of motor symptom onset (all *P*′*s* > 0.50). Accordingly, data were collapsed across these subgroups.

## 3. Results

### 3.1. Feasibility

We expected that 90% of participants would complete both the paper and the web-based questionnaire. This benchmark was surpassed with 100% completion. There was no difference between PD and HC in their ability to complete the online survey. Feedback indicated that the majority of participants rated the survey as easy to use and said that it could be completed within an hour (Table 2). The PD group took longer than the HC group to complete the questionnaire; the PD respondents had more questions to answer because there were items on PD-specific symptoms.

### 3.2. Visual and Visuospatial Abilities (PD and HC)

#### 3.2.1. Prevalence

**Figure 1:**
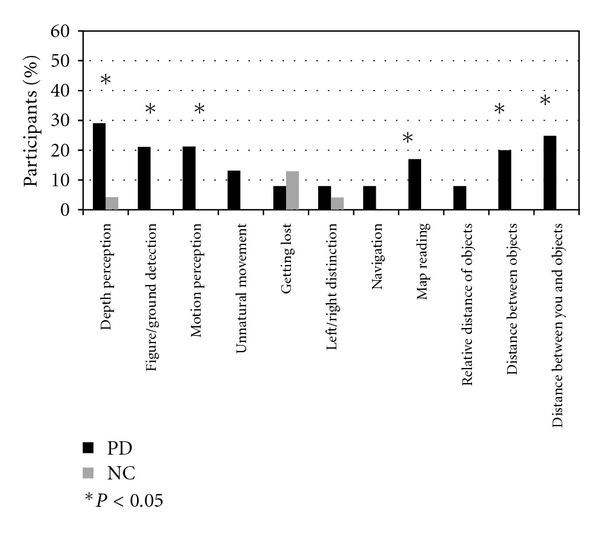
Percentage of PD and HC participants reporting impairment on the visual and visuospatial items from the web-based questionnaire.

Self-reported visual and visuospatial deficits were prevalent in this sample with 15 PD participants (63%) compared to six (26%) of HC reporting at least one deficit (*χ* ^2^ = 6.3, *P*′*s* < 0.01). A significantly greater percentage of PD than HC participants reported difficulties with depth perception, figure-ground discrimination, motion perception, map reading, and judging distances (all *P*′*s* < 0.05) (Figure 1).

#### 3.2.2. Reliability

**Figure 2:**
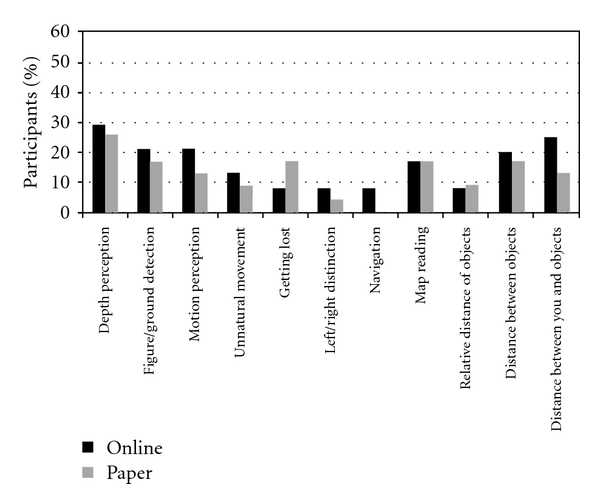
Percentage of PD participants reporting impairment on the visual and visuospatial items from the web-based versus paper-based questionnaires. There were no significant differences in item responding for the two formats (all *P*′s > 0.10).

For the PD group there was a significant association between the Visual and Visuospatial summary scores derived from the online and the paper survey (*r* = 0.71, *P* < 0.001), suggesting good consistency across formats (Figure 2). This association was also observed for the control group (*r* = 0.89, *P* < 0.001). The percentage of individuals reporting specific visuospatial difficulties did not significantly differ between formats (all *P*′*s* > 0.10).

**Validity of Web-Based Questionnaire (PD Only)**

With regard to construct validity, there was a highly significant negative association between the Visual and Visuospatial summary score from the web-based questionnaire and the *z*-score derived from performance on the objective visuospatial tests (composite score derived from Line Bisection, Road-Map, JLO) (*r* = −0.59, *P* < 0.003). That is, as self-reported visual and visuospatial impairments increased, performance on objective measures of visuospatial cognition was poorer. There were, however, no significant associations between the Visual and Visuospatial summary score derived from the questionnaire and the objectively based Vision *z*-score (composite score from FACT contrast sensitivity, backwards masking contrast sensitivity, coherent motion perception), or combined Visual and Visuospatial *z*-score (*P* > 0.30).

Exploratory analyses were conducted to determine if responses to specific visuospatial items on the questionnaire correlated with corresponding performance on the objective measures. In view of the potential number of comparisons, analyses were limited to correlations between the objective visuospatial measures that comprised the Visuospatial *z*-score (Line Bisection, Road Map, JLO) and construct-related web-based questions. To correct for multiple correlations (seven per visuospatial task), significant associations were defined as *P* ≤ 0.007 (alpha 0.05/7). Performance on each of Line Bisection and JLO was significantly correlated with a number of questions assessing self-reported high-order visuospatial abilities including navigation, map reading, and judging distances. Performance on the Road Map task was associated specifically with self-evaluated map reading abilities (Table 3). Overall, these findings provide support for the construct validity of the specific visuospatial items of left/right judgments, navigation, map reading, and estimating the distances between objects from the PD Vision Questionnaire.

### 3.3. Activities of Daily Living (PD)

#### 3.3.1. Reliability

Excellent reliability across measures was indicated by the strong correlation between the ADL summary scores derived from online and paper versions of the survey (*r* = 0.84, *P* < 0.001).

#### 3.3.2. Validity

Demonstrating construct validity of the ADL summary score, this score was significantly correlated with the ADL scale from the PDQ-39 (*r* = 0.44, *P* < 0.05) as well as the total PDQ-39 score (*r* = 0.49, *P* < 0.05).

### 3.4. Motor Symptoms (PD)

#### 3.4.1. Reliability

There was a strong association between the Motor summary scores derived from the online and paper formats (*r* = 0.80, *P* < 0.001).

#### 3.4.2. Validity

The self-reported Motor summary score and the UPDRS motor score were not significantly correlated (*r* = 0.11, *P* = 0.62). There was no association between the Motor summary score and the UPDRS motor score (*r* = 0.17, *P* = 0.45) even after conducting a partial correlation controlling for the duration of delay between UPDRS motor score rating and completion of the questionnaire (mean 7.4 months, SD 6.3 months). There was a significant correlation between the self-reported Motor summary score and the ADL section of the UPDRS (*r* = 0.57, *P* < 0.01), which evaluates functional and motor symptoms of PD through clinical interview with the participant.

## 4. Discussion

Our findings demonstrate the feasibility of using an online survey of visual and visuospatial abilities, ADL functioning, and motor symptoms by individuals with PD. Feasibility was also demonstrated in regard to self-evaluated visual and visuospatial abilities in healthy adults matched to the PD group for age, education, and other characteristics. Feedback from PD participants with mild to moderate disease severity and from HC indicated that the survey was easy to use and could be completed in a reasonable amount of time. All participants were able to complete both measures. The web-based assessment showed good reliability, comparable to other standard self-report measures of PD symptoms [29, 30]. Total Visual and Visuospatial summary scores did not differ between formats for the PD group (*P* > 0.1). The percentage of individuals with PD who reported difficulties on visuospatial items did not differ between formats (all *P*′*s* > 0.10). This result indicates that the formats have comparable sensitivity in regard to detecting visual and visuospatial impairments.

A greater percentage of the PD group than the HC group endorsed difficulties on most, but not all, of the visual and visuospatial items of the questionnaire. Non-endorsement of the “getting lost” item could indicate that individuals with PD did not have difficulties in these areas. Alternatively, the results could reflect reluctance on the part of PD respondents to admit difficulties in aspects of functioning that might be associated with serious outcomes such as the potential loss of a driver's license. There is also the possibility that these individuals had minimal exposure to situations requiring these abilities—for example, if they drove or went for walks only infrequently, possibly owing to PD-related motor impairment.

In general, self-reported visual and visuospatial deficits were prevalent in this PD sample, consistent with previous reports [1, 2]. More PD participants than HC endorsed visual difficulties, including in depth perception, motion perception, and figure ground discrimination, as well as in higher-order visuospatial cognition such as map reading and estimating distances. While PD-related deficits on objective measures of these specific abilities have been previously documented [4, 12, 31], it is striking that these impairments were severe enough to be noticed by a significant portion of the PD participants during everyday tasks requiring visuospatial abilities.

In the PD group, the number and severity of self-reported visuospatial impairments were associated with poorer performance on a composite measure of objective tests of higher-order visuospatial cognition. As participants reported greater difficulties with navigation, map reading, and judging distances, they performed more poorly on content-related neuropsychological measures of visuospatial cognition. It appears that individuals with PD, as assessed in this sample (nondemented, with mild to moderate motor severity), are sensitive to and can accurately report their visuospatial impairments. These findings point to an association between performance on laboratory-based measures of visuospatial cognition and visuospatial behavior in everyday life. The lack of association between objective measures of visual functioning and self-reported deficits could reflect either the participants' insensitivity to visual changes or the ability of our objective measures to detect visual changes before they are severe enough to impact performance of visually mediated tasks.

The severity of ADL impairment, as indexed by the online questionnaire, was significantly correlated with the degree of impairment that the participants with PD reported on the ADL scale as well as with the overall score of the PDQ-39, a commonly used measure of subjective quality of life [26]. These findings strongly suggest that web-based administration is a valid method of assessing difficulties with ADL functioning, which are frequently experienced by PD patients.

The Motor summary score of the web-based questionnaire was not correlated with the UPDRS motor score. It is possible that the restricted range of PD motor symptom severity in our sample (mild-moderate) might account for the absence of a significant association. One motivation for translating the questionnaire for online use is to capture the experience of PD participants with a broader range of disease severity than typically seen in research studies [12]. This includes individuals with more severe disease (H&Y 3+), which may prevent travel to a research setting or preclude lengthy in-laboratory assessments, as well as individuals with less severe disease (H&Y 1-2) who are still working and unable to take time out to participate. A second possible explanation for the lack of correlation between the survey's Motor summary score and UPDRS motor score is that in the former, the score is based on self-report whereas on the UPDRS the score is generated by an examiner. The lack of association between self- and examiner ratings of motor symptom severity also raises the possibility that our participants may not have viewed their motor symptom severity accurately, a finding that has been observed previously in patients with PD [32]. By contrast, the PD Vision Questionnaire Motor summary score was correlated with the UPDRS ADL score which, like the survey, reflects the participant's rather than the examiner's perspective. Participants may be more accurate in assessing their visuospatial and functional independence (ADLs), as these questions have better face validity than some of the motor symptoms assessed with the UPDRS. For example, unlike the spatial task of map reading, which patently assesses navigation abilities, many items on the motor scale of the UPDRS require evaluation of behaviors only rarely performed (e.g., tests of rapid alternating hand movements, force-induced loss of balance).

As the present study was conducted with a limited sample of participants, replication of our findings is needed to evaluate the stability of the reliability and validity associations. Larger samples would enable analysis by subgroups, which may be informative. For example, we recently reported that self-identified impairments in visual ADLs were more extensive in PD patients whose initial motor symptom was not tremor than in those whose initial symptom was tremor [33]. In particular, future work should focus on expanding the range of PD severity to include participants with milder and more severe motor impairment than were assessed here.

## 5. Conclusions

Internet-administered testing is a technology with the potential to increase participation in research and clinical trials by reducing the burden of engagement. Web-based administration may be especially useful in expanding the research pool of individuals with PD to include those who are not able to participate in laboratory-based research because of time constraints or mobility issues. The high rating of ease of accessibility and ease of completion of our online questionnaire lends confidence to the supposition that many PD patients with more severe disease may be able to complete this measure. The inclusion of patients with a broader range of PD symptom severity, both milder and more severe, would lead to a more accurate characterization of PD-related motor and nonmotor symptoms, particularly of understudied visual and visuospatial impairments.

## Figures and Tables

**Table 1:** Participant characteristics.

	PD (*n* = 24) Mean (SD)	HC (*n* = 23) Mean (SD)	*t* value^a^	*P*′*s* value
Age	65.7 (8.8)	66.7 (9.5)	0.39	ns
Education (yrs)	16.5 (2.3)	16.5 (2.4)	0.03	ns
Male : female	12 : 12	7 : 16	1.9^b^	ns
BDI	7.0 (5.2)	3.8 (4.2)^c^	2.3	0.03
BAI	8.0 (5.3)	2.4 (3.4)^c^	4.1	0.001
UPDRS—motor score	24.6 (9.8)	NA		
Disease duration (yrs)	7.0 (5.2)	NA		

^a^ *t*-values unless otherwise indicated; ^b^chi-squared value, ^c^df = 21; MMSE: modified Mini-Mental State Examination, scores converted to standard MMSE range; BDI: Beck Depression Inventory-II; BAI: Beck Anxiety Inventory; UPDRS: Unified Parkinson's Disease Rating Scale; NA: not applicable; ns: not significant.

**Table 2:** Questionnaire feedback collected online.

Feasibility questions	PD (*n* = 24)	HC (*n* = 23)
Difficult to complete? (1: not at all, 9: very much)	83% ratings of 1 or 2	91% ratings of 1 or 2

	34% indicated <30	96% indicated <30
Time to complete questionnaire (in minutes)	58% indicated 30–60	4% indicated 30–60
	8% indicated 60–90	

Instructions easy to understand? (1: not at all, 9: very much)	77% ratings of 8 or 9	91% ratings of 8 or 9

**Table 3:** Content validation of visuospatial items.

Visuospatial items from questionnaire	Visuospatial tasks		
	Landmark *N* = 24	JLO *N* = 23	Road Map *N* = 23
Getting lost	0.21	0.35	0.29
Left/Right decisions	0.54*	0.56*	0.41
Navigation	0.80*	0.56*	0.29
Map reading	0.86*	0.60*	0.55*
Judging objects in relation to each other	0.75*	0.54	0.27
Determining the distance between objects	0.55*	0.58*	0.48
Estimating distance between you and objects	0.39	0.43	0.26

*Pearson correlation *P*′*s* ≤ 0.007; Landmark: Landmark Test of Line Bisection; JLO: Judgment of Line Orientation.
